# Can mastalgia be another somatic symptom in fibromyalgia syndrome?

**DOI:** 10.6061/clinics/2015(11)03

**Published:** 2015-11

**Authors:** Meral Sen, Murat Ozgur Kilic, Ozlem Cemeroglu, Duygu Icen

**Affiliations:** I Turgut Ozal University, Department of General Surgery, School of Medicine, Ankara, Turkey; II Turgut Ozal University, Department of Physical Therapy, School of Medicine, Ankara, Turkey; III Hacettepe University, Department of Statistics, Ankara, Turkey

**Keywords:** Breast Pain, Fibromyalgia, Mastalgia, Breast Pain Questionnaire, Fibromyalgia Classification Criteria

## Abstract

**OBJECTIVE:**

The purposes of this study were to determine the coexistence of mastalgia and fibromyalgia, to investigate the effects of this combination on pain patterns, and to discuss the status of breast pain in the diagnostic algorithm of fibromyalgia syndrome.

**METHODS:**

Sixty-one female patients reporting breast pain during the last three months and 53 female patients diagnosed with fibromyalgia syndrome were enrolled in this study. The Breast Pain Questionnaire was administered to all participants in the mastalgia group and to those in the fibromyalgia syndrome group who had experienced mastalgia during the past three months. The patients in the fibromyalgia syndrome group were evaluated using the 2010 preliminary American College of Rheumatology classification criteria. All of the patients in the mastalgia group were evaluated for the diagnosis of fibromyalgia syndrome by a single physiatrist. The coexistence and pain patterns of mastalgia and fibromyalgia were assessed statistically.

**RESULTS:**

Approximately half of the patients with fibromyalgia syndrome (47.2%) reported having mastalgia at the time of admission and 37.7% of the patients with mastalgia met the diagnostic criteria for fibromyalgia syndrome. The patients with mastalgia in the fibromyalgia syndrome group had significantly higher total breast pain scores compared with the women in the mastalgia group. In addition, the patients with fibromyalgia syndrome in the mastalgia group had significantly higher Widespread Pain Index and Symptom Severity Scale scores than the patients with fibromyalgia syndrome.

**CONCLUSIONS:**

We suggest that mastalgia can be an aspect of the central sensitivity syndrome and can be added to the somatic symptoms of fibromyalgia.

## INTRODUCTION

Mastalgia, also referred to as mastodynia or breast pain, is a common compliant with a reported prevalence of 66% to 80% among women 1,2. Mastalgia is usually associated with benign breast conditions, such as fibrocystic breast disease, premenstrual syndrome and psychological disorders; less commonly, it is a sign of malignancy 3,4. Mastalgia is usually classified into two different types: cyclic and non-cyclic 5. Cyclic breast pain is the more common type; it is associated with menstrual periods and responds well to hormonal therapies 6. The non-cyclical type affects a small proportion of women without premenstrual exacerbations; its underlying physiopathology appears to be less clear than that of cyclic mastalgia. As a result, the etiopathogenesis of this commonly observed clinical entity is still unclear, but nutritional, hormonal and psychological causes are thought to play a role 7.

Most previous studies of mastalgia have focused primarily on its treatment modalities; however, a limited number of studies have investigated its psychological basis and its association with various psychological conditions or chronic pain syndromes 1,8. One of these is fibromyalgia syndrome (FMS), which is well known as a chronic musculoskeletal pain condition. Therefore, we aimed to explore the relationship between mastalgia and FMS, both of which are commonly found among female populations worldwide.

## MATERIALS AND METHODS

### Study design

Between July 2014 and December 2014, 114 consecutive women aged 18 years and older were included in this study, which took place in a tertiary-care university hospital. Sixty-one female patients presenting to our surgery department with breast pain of at least 3 months duration and 53 consecutive female patients admitted to the Physical Medicine and Rehabilitation Department with complaints of FMS were classified as the mastalgia group and the FMS group, respectively. The exclusion criteria were the presence of a previous organic breast disease, such as cancer, abscess, or a known breast pathology; previous breast surgery; pregnancy; lactation and an inability to complete the questionnaires.

The patients' age, educational status, marital status, duration of breastfeeding, menopausal status, hormone replacement therapy (HRT) use, employment status and comorbid diseases were recorded in detail.

All patients with complaints of breast pain were evaluated by the same surgeon and imaging methods such as mammography and/or ultrasonography were used when needed. To assess mastalgia, the Breast Pain Questionnaire (BPQ), a reliable and detailed tool, was administered to all of the participants in the mastalgia group and to those in the FMS group who reported having suffered from mastalgia in the 3 months prior to admission. This questionnaire provides information about the characteristics of breast pain, such as the pain's pattern, degree and relationship with menstruation. Furthermore, it is accepted as a quick assessment tool that physicians can use in daily practice 9. A final breast pain score (%totalBP) composed of four distinct scores (%Qtotal, %PPI, %VAS and %QOL, described below) was calculated for each patient with mastalgia. %Qtotal is the sum of the sensory and affective scores obtained from each sensory and affective descriptor. %PPI is the present pain index, %VAS is a score obtained using a visual analog scale and %QOL is a score based on quality of life questions. As a result, breast pain was classified into 3 severity groups based on the BPQ score: mild (0-100), moderate (101-200) and severe (>200).

The diagnosis of FMS was based on the 2010 preliminary American College of Rheumatology classification criteria 10. The criteria provide a current and detailed diagnostic tool consisting of two main parts: a widespread pain index (WPI) and a symptom severity scale score (SSS score). The WPI is a score between 0 and 19 related to specific painful areas on the body. The SSS score is based on the severity of fatigue, unrefreshing sleep and cognitive status, plus the presence of various somatic symptoms such as muscle weakness, nausea, vomiting, depression, insomnia, loss of appetite and thinking or memory problems.

An informed consent form was obtained from all participants. The study protocol was approved by the Medical Ethics Committee of Turgut Ozal University, Faculty of Medicine, Ankara, Turkey (permit number, date: 99950669/232, 30.06.2014).

### Statistical analysis

The Statistical Package for the Social Sciences (SPSS 21.0 software, Chicago, IL, USA) standard version was used for the data analyses. Descriptive analysis was performed for socio-demographic data and pain characteristics, and the statistical results were presented as the mean ± SD/percentages for continuous variables and number/percentage for categorical variables. The Mann Whitney U test and chi-square test were used to investigate the differences between the main groups and subgroups. The significance level was accepted as *p*<0.05.

## RESULTS

Sixty-one female patients with mastalgia aged 18 to 60 years and fifty-three female patients with FMS aged 18 to 48 years were included in this study. All of the patient characteristics are presented in Table 1. The mean ages of the mastalgia and FMS groups were similar (35.02 and 34.38 years, respectively) and the majority of the women in both groups were premenopausal (73.8% and 77.4%, respectively). Three patients in the mastalgia group and two patients in the FMS group received HRT for postmenopausal symptoms and there was no significant difference between the groups in this aspect (*p*=0.567). In addition, no significant differences were found between the two groups with respect to other patient characteristics, including educational status, marital status, employment status, duration of breastfeeding and presence of comorbid disease.

**Figure 1:**
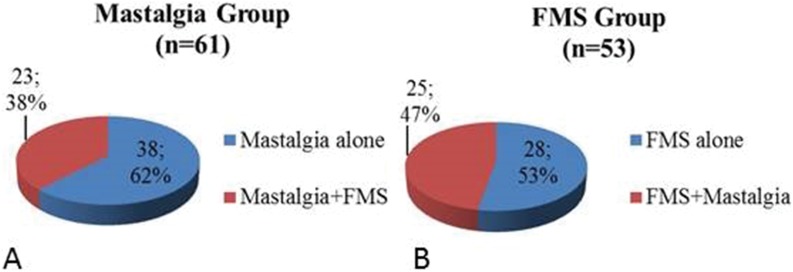
The number of patients diagnosed with fibromyalgia syndrome in the mastalgia group (A) and the number of patients with mastalgia in the FMS group (B).

Regarding the main outcomes of this study, 23 women (37.7%) were diagnosed with FMS in the mastalgia group according to the current diagnostic criteria and mastalgia was found in 25 patients (47.2%) in the FMS group (Figure 1).

All of the participants in the mastalgia group and the patients in the FMS group who suffered from mastalgia were compared in terms of the type of mastalgia, the severity of breast pain, and BPQ scores, including %Qtotal, %PPI, %VAS, %QOL, and %totalBP (Table 2). These two groups were similar with respect to age (*p*=0.790), educational status (*p*=0.743), marital status (*p*=0.771), employment status (*p*=0.626), menopausal status (*p*=0.822), duration of breastfeeding (*p*=0.392), presence of comorbid disease (*p*=1.000) and HRT (*p*=0.553). Cyclic mastalgia was the most frequent form in both groups. No significant difference was found between the women in the mastalgia group and those with mastalgia in the FMS group with respect to the type of mastalgia (*p*=0.321). Among the BPQ scores, the %Qtotal (*p*=0.029) and %VAS (*p*=0.010) were significantly higher among the subjects with mastalgia in the FMS group; however, there was no difference between the two groups in terms of %PPI (*p*=0.129) and %QOL (*p*=0.089). It was clear that the subjects with mastalgia in the FMS group had significantly higher totalBP scores compared with the patients in the mastalgia group (*p*=0.012). In addition, the severity of mastalgia, determined using the totalBP score, was similar for these two subgroups, with a *p*-value of 0.06.

All of the subjects in the FMS group and the participants who met the criteria for FMS in the mastalgia group were compared with respect to the WPI and SSS scores (Table 3). These two subgroups were similar in terms of age (*p*=0.412), educational status (*p*=0.591), marital status (*p*=0.172), employment status (*p*=0.179), menopausal status (*p*=0.752), duration of breastfeeding (*p*=0.986), presence of comorbid disease (*p*=0.648) and HRT (*p*=0.667). The mean WPI values for these subgroups were 8.43 and 9.87, respectively. The patients diagnosed with FMS in the mastalgia group had significantly higher a WPI compared with the women in the FMS group (*p*=0.024). In addition, the SSS scores of these two subgroups differed significantly (*p*=0.000). However, the scores for three SSS score subscales (fatigue [*p*=0.188], waking unrefreshed [*p*=0.314], and cognitive symptoms [*p*=0.159]) were statistically similar for the FMS group and the patients in the mastalgia group who met the criteria for FMS. In contrast, the somatic symptom score was significantly higher for the patients with FMS in the mastalgia group than for the patients in the FMS group (*p*=0.000).

## DISCUSSION

While mastalgia is a sign of an organic breast disease, it also has a possible psychological background that is not fully understood. In addition, mastalgia is known to be strongly associated with high stress levels 11. Unexplained pain syndromes, such as FMS and functional gastrointestinal disorders, including irritable bowel disease and dyspepsia, are also known to be stress-based diseases. Among these, irritable bowel syndrome has been found to be related to FMS, and both have a female predominance 12. Another study reported a strong link between patients with FMS and gastrointestinal disorders 13. Therefore, similar unknown mechanisms may play significant roles in the physiopathogenesis of mastalgia and similar stress-based diseases. Among those clinical entities, FMS has not been sufficiently investigated in terms of its association with mastalgia. However, the sociodemographic and clinical similarities between mastalgia and FMS suggest a possible relationship between them.

FMS, which is a common health problem, is defined as ongoing widespread pain of at least three months' duration combined with fatigue, sleep problems and various somatic symptoms. Although FMS is widely known as a rheumatic disease, it is also accepted as one of the central sensitivity syndromes 14. It mainly affects women and the associated chronic pain limits their quality of life. Therefore, FMS is a clinical condition that leads to significant socioeconomic problems worldwide. Additionally, patients with FMS often have various associated psychiatric disorders because of the physiological characteristics of the syndrome 15,16. In a study by Fietta et al., FMS was shown to be related to anxiety disorder and depression; up to 80% of FMS patients had anxiety or depression 17. Arnold also noted that psychiatric comorbidities negatively affect the severity and course of FMS 18. In our study, only two patients in the FMS group were taking psychiatric drugs at the time of admission; however, approximately one-quarter of the patients with FMS had a history of a psychiatric disorder at some point in their lives. Similarly, anxiety disorder and depression were found in a high percentage of the mastalgia patients in a study by Colegrave 8. To our knowledge, the first comprehensive clinical study on the association of mastalgia with psychiatric conditions was reported by Preece et al. in 1978. He found that treatment-resistant patients with breast pain had higher scores for anxiety and depression 19. Later, a 1993 study by Jenkins et al. showed that 21 out of 25 patients with treatment-resistant breast pain met the criteria for any psychiatric diagnosis. The authors recommended psychiatric evaluation and antidepressant therapy for this group of mastalgia patients based on their findings 20. Similarly, 13 out of 61 mastalgia patients in our study had been previously treated for a psychiatric disorder. To the best of our knowledge, only two clinical studies on the relationship between mastalgia and FMS have been reported to date 1,21. One is the 2006 study by Johnson et al, which demonstrated the association between mastalgia and various chronic pain disorders, including irritable bowel syndrome, chronic pelvic pain and FMS 1. However, the severity of mastalgia was not evaluated using a current scoring system; instead, mastalgia was only classified as frequent or infrequent. Additionally, the authors did not determine the prevalence of mastalgia in patients with FMS. Despite these limitations, one of the main outcomes of their study was the finding of a possible association between a common form of mastalgia and unexplained pain syndromes. The second study to demonstrate a link between mastalgia and FMS was reported by Genc et al. in 2011 21. In that study, the BPQ was used as a relatively objective assessment tool for evaluating mastalgia. We also used this questionnaire to evaluate breast pain; however, our study was differentiated by its use of new diagnostic criteria for FMS. In contrast, the authors of the previous study used the 1990 American College of Rheumatology classification criteria, the Fibromyalgia Impact Questionnaire and Short Form-36 to diagnose and assess FMS. The 1990 criteria were based on a count of tender points at different body sites. In 2010, these criteria were abandoned in favor of a new diagnostic classification system that placed increased emphasis on the patient symptoms published by The American College of Rheumatology 10. This current diagnostic tool includes both the WPS and SSS scores, which allows physicians to assess various polysymptomatic conditions, including fatigue, sleep status, cognitive status and various somatic symptoms. Therefore, the 2010 preliminary criteria provide more information about symptom variability in patients with FMS and are more sensitive than the 1990 criteria 22. In the study by Genc et al., two distinct clinical entities, mastalgia and FMS, were found to frequently coexist. According to that study's findings, 36% of the mastalgia patients met the criteria for FMS, while 42% of the patients with FMS suffered from mastalgia. We found a higher association between mastalgia and FMS than the previous authors did. From our point of view, the higher rates of FMS observed in our study were a consequence of the new FMS classification system. Approximately half of the patients with FMS in our study had mastalgia; this ratio may be comparable to that of the general population because the majority of women suffer from breast pain at some point in their lives 23. It is important to note that patients undergoing HRT were not removed from our study. In fact, HRT is a known cause of mastalgia, but it was not used as an exclusion criterion because the number of patients undergoing HRT was very low in both groups and had no effect on the statistical results.

The total breast pain scores of the patients with mastalgia in the FMS group were significantly higher than those of the women in the mastalgia group. The scores for the other scale, severity of breast pain, did not reach statistical significance, but their *p*-value of 0.060 was very close to the significance level. However, Genc et al. found that these scores were similar for the two groups 21. These two conflicting results may be the result of the limited number of patients enrolled in the study; however, the coexistence of these two disorders seems to aggravate the severity of mastalgia. Furthermore, these results are of great importance to the mastalgia treatment approach. It is well known that many hormonal and non-hormonal agents have been used in mastalgia treatment 4; however, none of them offers a total cure. In addition, widespread pain assessment is usually neglected in mastalgia patients and may contribute to the failure of mastalgia treatment. Therefore, patients with mastalgia should be evaluated for the presence of FMS to increase the likelihood of successful pain treatment.

The other main finding of our work is the high prevalence of FMS in mastalgia patients (37.7%) compared with the general population. This result may reveal a common etiopathogenic link between mastalgia and FMS. In addition, when the FMS classification scores of the groups were analyzed, the WPI and SSS scores differed significantly between the FMS group and the patients with FMS in the mastalgia group. Additionally, a statistically significant difference was found between these groups regarding their somatic symptom scores. The statistically higher scores of the patients with FMS in the mastalgia group may indicate that mastalgia could be included among the somatic symptoms in the diagnostic algorithm for FMS. Additionally, these findings can help to extend the current FMS concept and the consideration of mastalgia could change our approach to FMS treatment. Further large-scale clinical studies could help to clarify the association between mastalgia and the success of pain management in FMS.

This study showed that the coexistence of mastalgia and FMS is more frequent than previously estimated, suggesting that these two disorders could share some unknown common mechanisms in their etiopathogenesis. Additionally, mastalgia could be a part of central sensitivity syndrome and could be included among the somatic symptoms in the fibromyalgia criteria based on its high prevalence in patients with FMS. Therefore, patients with FMS should also be asked about the presence of mastalgia during routine examinations.

## Figures and Tables

**Table 1:** Comparison of the patient characteristics of the two groups.

	Mastalgia group (n=61)	Fibromyalgia syndrome group (n=53)	*p*
Age (years)	35.02 ± 9.90	34.38 ± 8.20	0.930
Educational status			0.425
Primary/secondary school	17 (27.9%)	20 (37.7%)	
High school	26 (42.6%)	22 (41.5%)	
University	18 (29.5%)	11 (20.8%)	
Marital status			0.535
Single	12 (19.7%)	11 (20.8%)	
Married	49 (80.3%)	42 (79.2%)	
Employment status			0.507
Housewife	37 (60.7%)	33 (62.2%)	
Employed	24 (39.3%)	20 (37.8%)	
Menopausal status			0.777
Premenopausal	45 (73.8%)	41 (77.4%)	
Perimenopausal	9 (14.8%)	8 (15.1%)	
Postmenopausal	7 (11.5%)	4 (7.5%)	
Duration of breastfeeding (mo.)	19.87 ± 20.40	24.17 ± 21.04	0.289
Comorbid disease	4 (6.5%)	3 (5.6%)	0.587
HRT	3 (4.9%)	2 (3.7%)	0.567

Data are presented as the mean ± SD for age and duration of breastfeeding; n (%) for other variables. mo.: months, HRT: hormone replacement therapy.

**Table 2:** Comparison of the breast pain patterns of the patients with mastalgia in the two groups.

	Mastalgia group (n=61)	Patients with mastalgia in the fibromyalgia syndrome group (n=25)	*p*
Age (years)	35.02 ± 9.902	33.76 ± 8.227	0.790
Type of breast pain			0.321
Cyclic	42 (68.9%)	14 (56.0%)	
Non-cyclic	19 (31.1%)	11 (44.0%)	
BPQ scores			
%Qtotal	30.07 ± 15.788	42.32 ± 23.167	****0.029****
%PPI	16.75 ± 7.824	19.52 ± 7.784	0.129
%VAS	51.80 ± 18.029	62.00 ± 13.540	****0.010****
%QOL	33.21 ± 20.239	40.40 ± 19.333	0.089
%totalBP	131.90 ± 56.334	164.16 ± 53.330	****0.012****
Severity of mastalgia			0.060
Mild	23 (37.7%)	3 (12.0%)	
Moderate	27 (44.3%)	15 (60.0%)	
Severe	11 (18.0%)	7 (28.0%)	

Data are presented as the mean ± SD for age and BPQ scores; n (%) for other variables.

**Table 3:** Comparison of the patients with fibromyalgia syndrome in the two groups.

	Fibromyalgia syndrome group (n=53)	Patients with fibromyalgia syndrome in the mastalgia group (n=23)	*p*
Age (years)	34.38 ± 8.200	36.61 ± 9.389	0.412
WPI	8.43 ± 1.896	9.87 ± 2.912	**0.024**
SSS score	6.34 ± 1.568	8.00 ± 2.276	**0.000**
Fatigue score	2.06 ± 0.745	2.30 ± 0.635	0.188
Waking unrefreshed score	2.04 ± 0.678	2.17 ± 0.887	0.314
Cognitive symptoms score	1.25 ± 0.757	1.48 ± 0.730	0.159
Somatic symptoms score	1.02 ± 1.009	2.04 ± 0.878	**0.000**

Data are presented as the mean ± SD for all variables.
